# Iatrogenic Direct Carotid-cavernous Fistula Following Mechanical Thrombectomy: A Case Report and Review of the Literature

**DOI:** 10.7759/cureus.7524

**Published:** 2020-04-03

**Authors:** Dallas L Sheinberg, Marie-Christine Brunet, Stephanie H Chen, Evan Luther, Robert M Starke

**Affiliations:** 1 Neurological Surgery, University of Miami Miller School of Medicine, Miami, USA

**Keywords:** carotid cavernous fistula, ccf, thrombectomy, endovascular, complications, stroke

## Abstract

A carotid-cavernous fistula (CCF) is an abnormal connection between the arteries and veins of the cavernous sinus. Iatrogenic CCFs have been described as potential complications following aneurysm coiling, balloon angioplasty, and transsphenoidal surgery. In this case report, we describe a rare case of an iatrogenic direct CCF following mechanical thrombectomy (MT) for acute ischemic stroke. A 78-year-old female presented to an outside hospital with a new onset of right-sided weakness and aphasia and underwent emergency MT for a left middle cerebral artery (MCA) occlusion. The procedure was complicated by iatrogenic injury to the left cavernous internal carotid artery (ICA), which resulted in a direct high-flow CCF. The patient was transferred to our hospital and the fistula was closed with transarterial coils. Ten days later, she returned with diplopia and cranial nerve VI palsy due to residual pseudoaneurysm and was treated with a flow-diverting stent. On follow-up, the patient was neurologically intact and imaging showed no residual fistula. As the frequency of MTs performed for acute ischemic stroke continues to rise, neurointerventionalists should be aware of this potential rare complication and be prepared to manage patients who develop symptomatic CCF.

## Introduction

A carotid-cavernous fistula (CCF) is an abnormal connection between arteries and veins within the cavernous sinus. CCFs have been classified according to hemodynamic properties (high-flow vs. low-flow), etiology (spontaneous vs. traumatic/iatrogenic), and anatomical feeders (direct vs. indirect). A direct high-flow CCF results from a tear in the wall of the cavernous internal carotid artery (ICA), thereby shunting blood from the ICA to the cavernous sinus. Iatrogenic high-flow CCFs have been described as a complication in approximately 0.8% of endovascular interventions involving the anterior circulation including elective aneurysm coiling, intracranial balloon angioplasty, and mechanical thrombectomy (MT) [1-4]. 

The number of MTs performed in the United States for acute ischemic stroke is steadily rising due to the mounting evidence of its benefit in select patients, as it can limit disability from stroke even up to 24 hours [5-9]. However, in rare circumstances, endothelial injury by microwire manipulation or pulling of the stent retriever through the cavernous ICA during MT can cause vessel injury. In this article, we present a rare case of an iatrogenic direct CCF following MT for acute ischemic stroke and engage in a review of the related literature.

## Case presentation

A 78-year-old female with a past medical history of hypertension and breast cancer presented to an outside hospital with aphasia and right hemiplegia after a syncopal episode with a National Institutes of Health Stroke Score (NIHSS) of 18. The patient underwent an emergency MT for a left middle cerebral artery (MCA) occlusion, and successful revascularization was achieved after two passes with a stent retriever. The procedure was complicated by iatrogenic injury of the left cavernous ICA, resulting in a direct high-flow CCF. The patient was referred to our center a few days later after an unsuccessful attempt to occlude the fistula. On arrival at our center, the patient had fully recovered from her previous left MCA stroke with an NIHSS of 0 and no neurologic deficit. She was on a daily antiplatelet regimen of aspirin 81 mg. The patient was asymptomatic from the direct CCF, and an ophthalmology examination revealed normal extra-ocular movements, normal visual acuity, and normal ocular tension (measured by tonometry; Tono-Pen, Reichert Technologies, Depew, NY) in both eyes (right 16 mm Hg and left 17 mm Hg).

**Figure 1:**
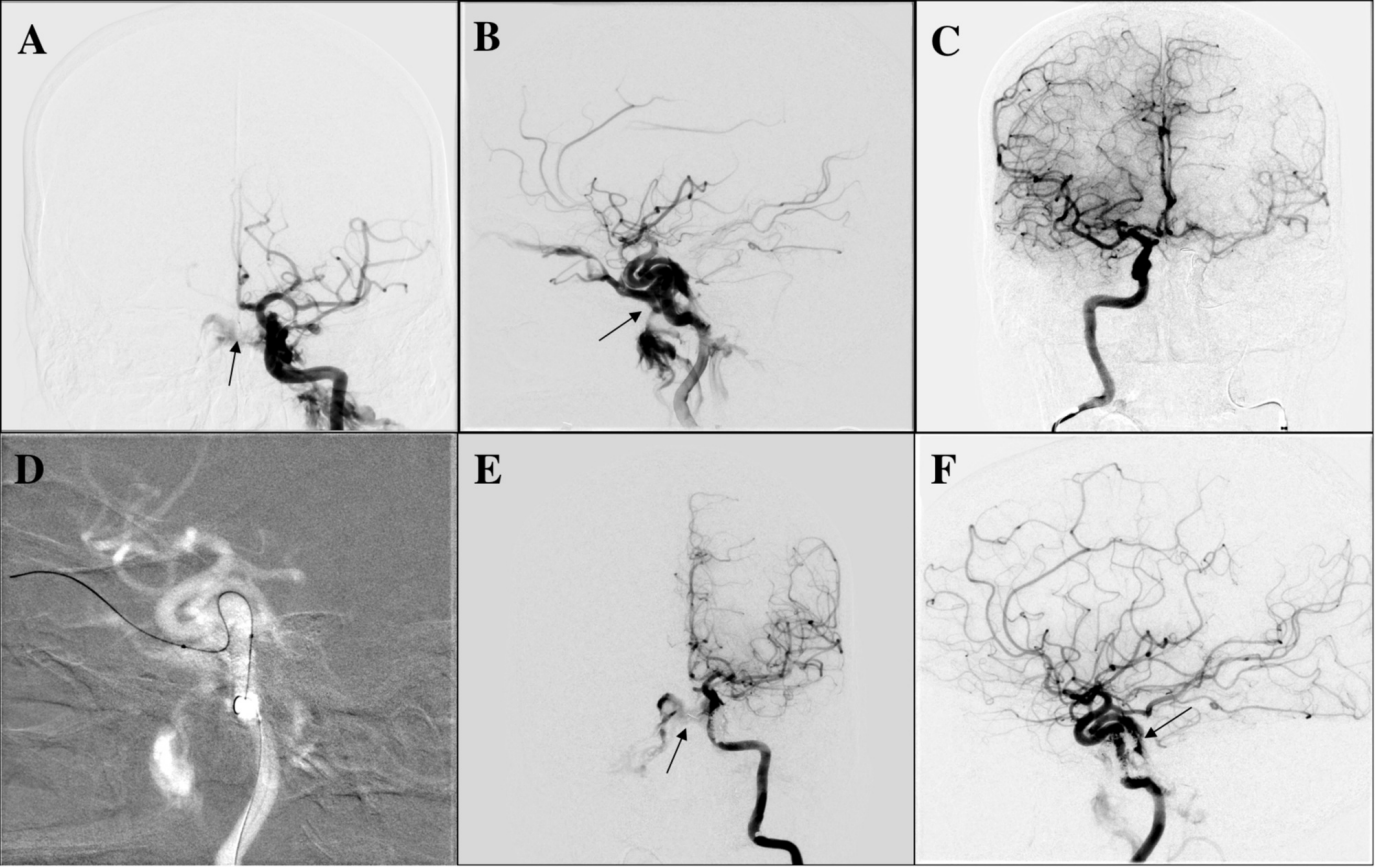
Diagnostic cerebral angiogram and transarterial embolization of the left CCF (A) AP and (B) lateral view of left ICA injection demonstrating direct/high-flow left CCF (arrow). The images show venous drainage into bilateral cavernous sinus, left superior ophthalmic vein, and left pterygopalatine plexus; (C) balloon test occlusion of left ICA with TransForm balloon of 4 x 10-mm and injection from right ICA. The image shows venous delay in left hemisphere of 1.8 seconds; (D) supra-selective catheterization of the fistula point and distal left superior ophthalmic vein with synchro soft microwire and headway duo microcatheter; (E) AP and (F) lateral view of left ICA injection after coiling showing residual CCF draining into a posterior venous pouch, bilateral cavernous sinus, and left pterygopalatine plexus (arrow). The images show neither ophthalmic venous drainage nor cortical venous drainage AP: anteroposterior; ICA: internal carotid artery; CCF: carotid-cavernous fistula

After reviewing the angiographic images from the outside hospital, the decision was made to proceed with diagnostic cerebral angiogram and transarterial embolization of the left CCF (Figure 1). After completing a full six-vessel diagnostic cerebral angiogram, balloon test occlusion of left ICA was done using a 4 x 10-mm compliant TransForm balloon (Stryker Corporation, Kalamazoo, MI) that was inflated across the injured segment of left cavernous ICA. Injection from the right internal carotid was done and demonstrated a “grey zone” result with a venous delay of 1.8 seconds between the two hemispheres. Given the balloon test occlusion result, transarterial CCF embolization was favored over therapeutic ICA sacrifice as the treatment for this specific case. Thereafter, the microcatheter was navigated transarterially through the hole in the left ICA into the left cavernous sinus and left superior ophthalmic vein (SOV). After the satisfactory positioning of microcatheter in the distal left SOV, coil embolization was performed in a retrograde fashion from the SOV toward the cavernous sinus near the communication with the injured cavernous ICA. We were unable to deploy the final coil at the fistulous point as it was protruding into the parent vessel (ICA) even with balloon assistance. Minimal slow residual shunting was seen in a posterior venous pouch draining into the pterygopalatine plexus at the conclusion of the procedure. No residual ophthalmic venous drainage was seen. There were no complications, and the patient was discharged neurologically intact the following day on antiplatelet therapy with aspirin 325 mg and Plavix 75 mg in preparation for further treatment of residual fistula with flow diverter embolization.

**Figure 2:**
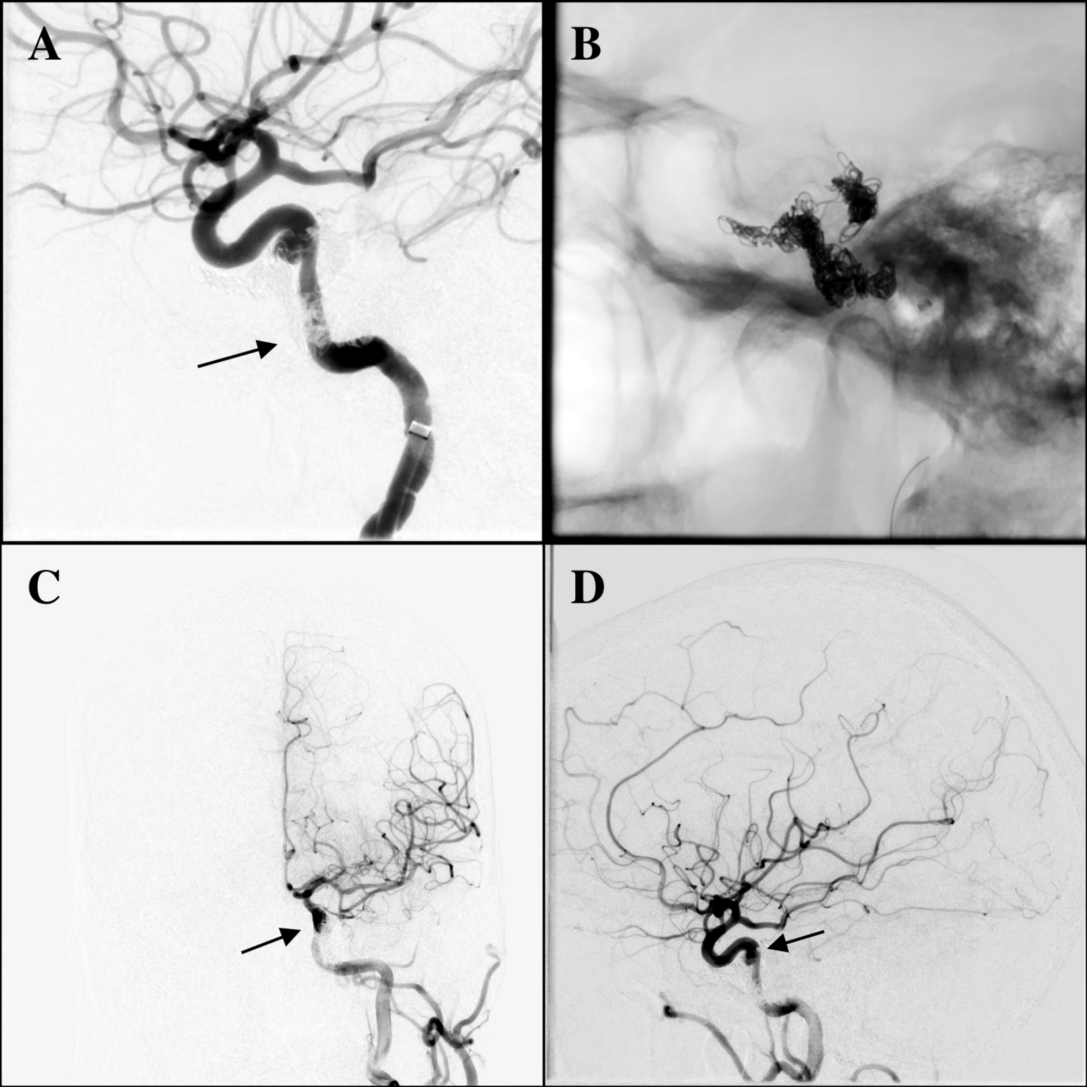
Angiogram of left ICA before pipeline treatment; pipeline deployment; and left ICA injection after pipeline treatment (A) Follow-up angiogram of left ICA 10 days later with no residual left CCF (arrow). The image shows no early vein or venous pouch opacification. However, it shows residual pseudoaneurysm neck of left cavernous ICA at the previous shunting point location. (B) Pipeline deployment in left cavernous ICA covering pseudoaneurysm. (C) Final AP and (D) lateral view of left ICA injection after pipeline treatment (arrow) AP: anteroposterior; ICA: internal carotid artery; CCF: carotid-cavernous fistula

The patient returned 10 days later with diplopia, headaches, and tinnitus. Physical exam revealed a lateral gaze palsy in her left eye. On angiogram, no residual CCF was visualized; however, a residual pseudoaneurysm was observed in the left cavernous ICA at the previous location of the fistula shunting point (Figure 2). Considering the initial mechanism of injury and the new symptoms experienced by the patient, the decision was made to proceed with pipeline embolization of this residual ICA pseudoaneurysm. A coaxial system of an 088” ID guide catheter, an intermediate catheter (060” Syphontrak, Codman Neuro, Raynham, MA), and 027” Phenom microcatheter (Medtronic, Minneapolis, MN) were placed into the left supraclinoid ICA, and a Pipeline Flex 3.5 mm x 16 mm (Medtronic, Minneapolis, MN) device was deployed to cover the neck of the left cavernous ICA pseudoaneurysm. The procedure was uncomplicated and the patient remained asymptomatic at the six-month follow-up. Follow-up CTA showed no residual fistula or aneurysm.

## Discussion

The Barrow classification categorizes CCFs into four types: Type A fistulas are direct connections between the ICA and cavernous sinus, and Type B, C, and D fistulas are all indirect dural shunts from meningeal branches of the ICA, external carotid artery (ECA), or both, respectively [10]. The etiology of direct Type A CCFs typically includes blunt or penetrating trauma, ICA aneurysm rupture, Ehlers-Danlos syndrome type IV, or rarely, iatrogenic interventions [11]. Additionally, CCFs are more frequently reported in female patients, suggesting a differential vascular weakness between the sexes [1]. The patient in our case was a female without any known vascular predisposing risk factors presenting with iatrogenic direct CCF.

Type A fistulas often present with severe symptoms due to the high-flow fistulous connection between the ICA and cavernous sinus. Patients may present with chemosis, orbital congestion, proptosis, double vision, cranial nerve dysfunction, ocular bruit, and increased intraocular pressure leading to central retinal vein occlusion or optic neuropathy [11]. Cranial nerve VI palsies frequently occur due to the sixth nerve location adjacent to the ICA, compared to the more lateral location of other cranial nerves within the cavernous sinus. Treatment of these fistulas by endovascular occlusion of the fistula usually resolves with the rapid resolution of symptoms.

To our knowledge, there are only three other studies in the literature describing iatrogenic direct CCF following MT (Table 1). Alan et al. reported three cases of Type A CCFs that occurred following MT for acute ischemic stroke [3]. In the first case, the patient presented with a tandem occlusion of the right ICA and MCA that was treated with stenting of the cervical ICA and three passes of the stent retriever with local aspiration. A small CCF was noted postoperatively, however, the patient had a poor neurologic outcome and treatment was not pursued. The patient’s CCF remained asymptomatic after 1.5 years of follow-up. In the second case, a CCF was noted in a patient after two passes of stent retriever thrombectomy with local aspiration for a left ICA terminus occlusion. The CCF was managed conservatively, and the patient remained asymptomatic at the four-month follow-up. In the third case, a patient with a tandem right ICA and MCA occlusion underwent thrombectomy with two passes of the stent retriever. However, overnight, there was complete re-occlusion of the ICA, which required angioplasty and stenting of the cervical carotid, stenting of the cavernous/lacerum carotid, and manual aspiration thrombectomy of an iatrogenic right MCA occlusion. A CCF was observed at the conclusion of the procedure but was not treated. Two weeks after discharge, the patient developed proptosis, chemosis, and a right cranial nerve VI palsy. Embolization was performed via a transvenous inferior ophthalmic vein approach with a complete resolution of symptoms. Similarly, Matsumoto et al. reported a case of MT for a right M1 proximal occlusion where strong resistance was felt during the first pass of stent retriever withdrawal [12]. This resulted in a stretch of the cavernous ICA by the anchoring of the balloon and stent retriever, resulting pullout injury to the meningohypophyseal trunk and CCF. Symptoms initially related to the CCF included hyperemia and chemosis. The CCF was treated three weeks after thrombectomy with transvenous coil embolization via an inferior petrosal sinus approach. Postoperatively, the ocular symptoms disappeared and there was no ocular movement disorder at the three-month follow-up. 

**Table 1:** Literature review of iatrogenic direct CCF following mechanical thrombectomy ICA: internal carotid artery; MCA: middle cerebral artery; M1: MCA segment 1; CN VI: cranial nerve six; CCF: carotid-cavernous fistula; NA: not available

Literature review of iatrogenic direct CCF following mechanical thrombectomy						
Author	Alan et al. [3]			Akpinar et al. [2]	Matsumoto et al. [12]	Current study
	Case 1	Case 2	Case 3			
Site of occlusion	Tandem right ICA terminus and MCA	Left ICA terminus to left M1	﻿Right ICA (petrous, lacerum, proximal cavernous segment), proximal right M1	Right distal ICA and right MCA	Right M1	Left MCA
Number of passes	﻿1	2	1	NA	1	2
Procedure	Stent retriever thrombectomy with local aspiration; stenting of cervical carotid	Stent retriever thrombectomy with local aspiration	﻿Stenting of the cervical/cavernous/lacerum carotid; manual aspiration thrombectomy	Stent retriever thrombectomy with local aspiration	Stent retriever thrombectomy	Stent retriever thrombectomy with local aspiration
Initial CCF symptoms	Asymptomatic	Asymptomatic	Right CN VI palsy, proptosis, chemosis (asymptomatic after 2 weeks)	NA	Chemosis, hyperemia	Asymptomatic
Management of CCF	Conservative	Conservative	Embolization via transvenous inferior ophthalmic vein approach (after initial conservative management)	Conservative (ICA was not recanalized)	Embolization via transvenous inferior petrosal sinus approach (three weeks post- thrombectomy)	Embolization via transarterial approach to superior ophthalmic vein; 10 days later, pipeline embolization of residual pseudoaneurysm
Follow-up	1.5 years: asymptomatic	4 months: asymptomatic	2.5 years: asymptomatic	Died 2 days postoperatively	3 months: asymptomatic	6 months: asymptomatic

In a retrospective analysis, Akpinar et al. assessed complications of 28 consecutive stroke patients and reported one case of CCF following MT of a right ICA terminus occlusion [2]. The ICA terminus was not recanalized and the patient died a few days later. 

In all the patients who developed a CCF after thrombectomy, a stent retriever was used and multiple passes were required. Both aspiration and stent retriever thrombectomy have been shown to cause vessel wall damage in animal models. However, stent retriever thrombectomy appears to cause more aggressive damage with areas of complete denudation of the endothelium after just one pass on histology [13]. Repeated passes of the stent retriever may further increase the acute damage, weakening the vessel wall, and likely increasing the risk for subsequent perforation or CCF.

In general, Type A traumatic CCFs are unlikely to close spontaneously, and there is a risk of progression with approximately 20% of patients requiring emergency treatment secondary to acute decreases in visual acuity, increases in intraocular pressure, or intracranial hemorrhage [14]. However, the natural history of iatrogenic Type A fistulas by the navigation of the microwire or stent retriever remains unclear. While two patients in the Alan et al. series remained asymptomatic on short-term follow-up, only 25% of patients had spontaneous resolution of iatrogenic CCF in a series examining all neurointerventional procedures [1]. Further studies are necessary to determine if the decision to treat thrombectomy-associated CCFs should be delayed until the patient becomes symptomatic or dependent on particular qualities of the fistula. 

Treatment options for CCF include surgery, radiosurgery, or endovascular interventions. In cases of direct CCF, the first-line treatment is endovascular occlusion, which may be accomplished using stand-alone coiling, stent-assisted coiling, detachable balloons, liquid embolic agents, or a combination of these strategies [4,11,15]. After CCF occlusion, pseudoaneurysm formation may occur and is typically managed conservatively unless the patient is symptomatic. The CCF in our case was treated by coil embolization via a direct transarterial approach followed by flow-diverting stent placement for a symptomatic residual pseudoaneurysm of the ICA.

## Conclusions

We presented a rare case of iatrogenic direct CCF following MT for acute ischemic stroke. As the popularity of endovascular treatment for acute ischemic stroke continues to increase, it is important for neurointerventional operators to be aware of potential iatrogenic direct CCF complications, particularly in cases requiring multiple passes of a stent retriever, angioplasty, or stent placement.
